# Updates on CAD risk assessment: using the coronary artery calcium score in combination with traditional risk factors

**DOI:** 10.1186/s43044-025-00608-4

**Published:** 2025-01-23

**Authors:** Kiara Rezaie-Kalamtari, Zeinab Norouzi, Alireza Salmanipour, Hossein Mehrali

**Affiliations:** 1 Rajaie Cardiovascular, Medical and Research Institute, Valiasr Ave, Hashemi Rafsanjani (Niayesh) Intersection, Tehran, Iran; 2 Rajaie Cardiovascular, Medical & Research Center, School of Medicine, Iran University of Medical Sciences, Tehran, Iran

## Abstract

**Background:**

Coronary artery disease (CAD) is the third leading cause of death worldwide, so prevention and early diagnosis play important roles to reduce mortality and morbidity. Traditional risk-score assessments were used to find the at-risk patients in order to prevent or early treatment of CAD. Adding imaging data to traditional risk-score systems will able us to find these patients more confidently and reduce the probable mismanagements.

**Main text:**

Measuring the vascular calcification by coronary artery calcium (CAC) score can prepare valuable data for this purpose. Using CAC became more popular in recent years. The most applicable method to evaluate CAC is Agatston scoring using computed tomography (CT) scanning. Patients are classified into several subgroups: no evidence of CAD (score 0), mild CAD (score 1–10), minimal CAD (score 11–100), moderate CAD (score 101–400), and severe CAD (score > 400) and higher than1000 as the extreme risk of CVD events.

**Conclusions:**

CAC assessment was recommended in the patients older than 40 years old with CAD risk factors, the ones with stable angina, borderline-to-intermediate-risk group, etc. According to the results of the CAC the patients may be candidate for further evaluation for needing revascularization, medical treatment, or routine follow-up. Adding artificial intelligence (AI) to CAC will prepare more data and can increase the reliability of our approach to the patients promising a bright future to improve this technology.

## Background

### Atherosclerotic coronary artery disease prevalence and prevention

Coronary artery disease (CAD) is the third leading cause of death worldwide, cutting about 17.8 million lives short annually. The good news is, however, that this monstrous disease is preventable since coronary atherosclerosis is amenable to primary prevention [1–4].

To prevent atherosclerotic cardiovascular disease (ASCVD), the Framingham Heart Study constructed a scaffold for its risk stratification that is still in wide use for individual overall risk assessment [5–8]. The pooled cohort equation (PCE) uses age, gender, total cholesterol, low- and high-density lipoprotein, systolic blood pressure (BP), diabetes, smoking, and a history of treatment for high BP to calculate the 10-year risk for ASCVD [9]. Nonetheless, strong dependency on gender and age has raised questions about the capability of PCE to identify low-risk individuals, especially middle-aged persons, elderly men, and patients with different ethnicities [10–13]. To prevent risk overestimation, researchers have introduced markers that discriminate low-risk individuals mislabeled as “at risk” based on traditional risk-score systems. This discrimination is crucial to clinical decision-making and eventually reducing unnecessary treatments [14–16].

Several studies have regarded a zero coronary artery calcium (CAC) score, commonly termed “the power of zero,” as the most potent negative risk marker in identifying low, absolute risk individuals [16–20]. Despite the strong negative predictive value of a CAC score of zero, one-fourth to one-third of CVD events occur in this group. Notably, the prevalence of traditional and potentially modifiable CVD risk factors is higher in patients with a CAC score of zero who experience CVD events [21]. On the other hand, the predictive capability of the CAC score surpasses the Framingham risk score (FRS) or the 2013 American College of Cardiology/American Heart Association (ACC/AHA) pooled cohort equations in predicting ASCVD events when used exclusively [22, 23]. Furthermore, the CAC score has incremental value when combined with traditional risk assessment methods [24, 25], hence the significance of a risk stratification system based on both traditional risk factors and imaging findings.

## Main text

### The CAC score method

**Fig. 1:**
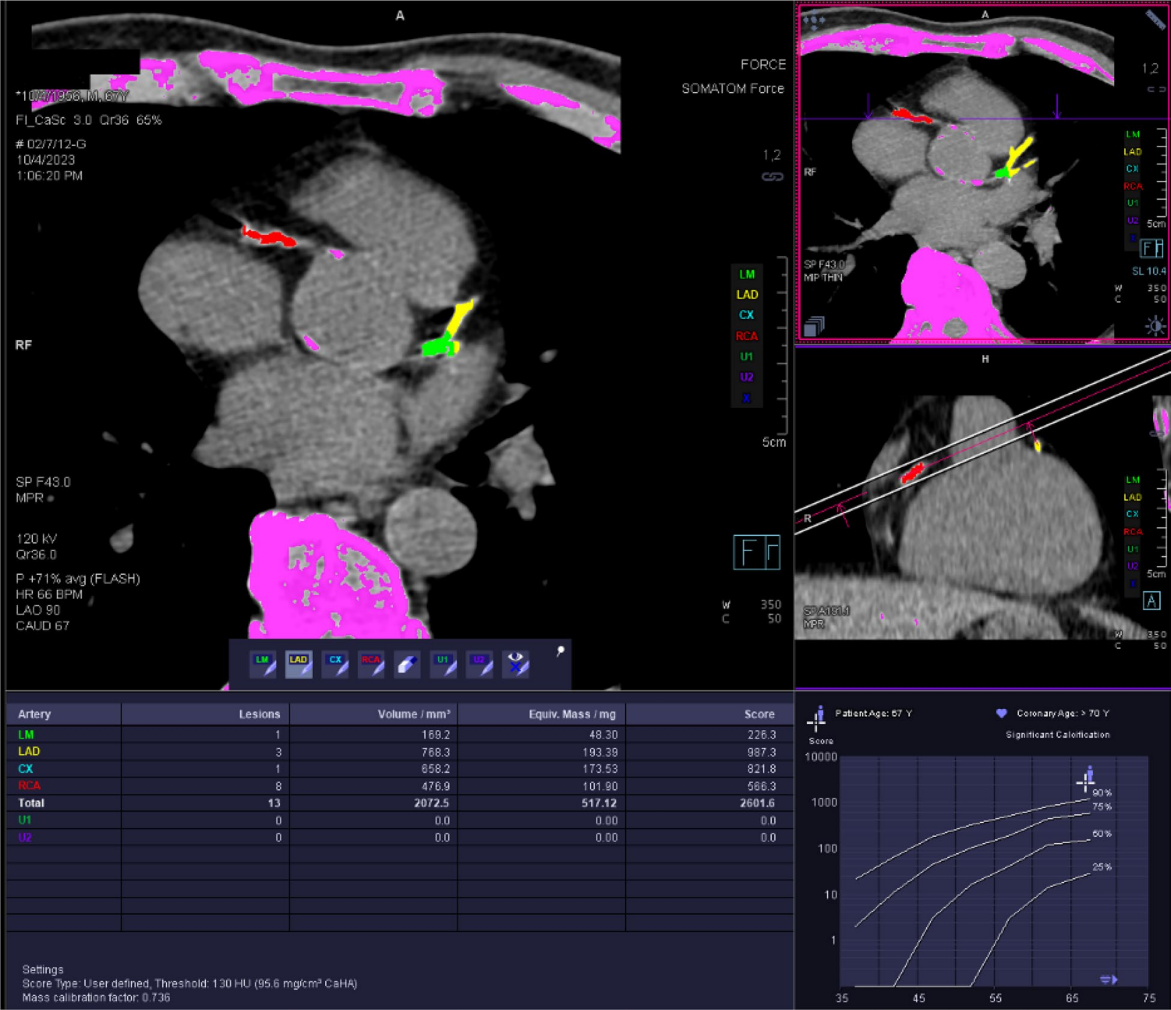
An example of the CAC result: A total score is 2601 with 13 lesions. The maximum calcium burden was found in LAD with CAC of 987. CAC: Coronary artery calcium, LAD: Left anterior descending arter

Vascular calcification is a surrogate for atherosclerotic changes [26]. Imaging techniques can effectively detect and measure vascular calcification, making imaging an effective screening tool for atherosclerotic changes [27]. Several methods are available for CAC scoring, the most applicable of which is Agatston scoring. This method uses computed tomography (CT) scanning to measure the coronary calcium in the heart arteries according to the weighted density score given to the highest attenuation value multiplied by the area of the calcification speck. The total calcium score is defined as the score of every calcified speck summed up [28] (Fig. 1).

Ideally, CAC score measurement is performed on dedicated electrocardiogram (ECG)-gated non-contrast-enhanced cardiac CT machines with a slice thickness of 2.5–3 mm, a tube voltage of 120 kV, and an arbitrary milliampere based on the patient’s body habitus [4, 29]. Still, several studies have shown promising results with non-ECG-gated chest CT scans to calculate the CAC score [30–33]. Filtered back-projection is commonly implemented for image reconstruction; nevertheless, validated iterative or model-based reconstruction algorithms can also be utilized [34]. Iterative reconstruction models offer several advantages, including enhanced image quality, reduced effective dose, and augmented small calcification detection, justifying the recent trend toward the use of iterative reconstruction models [35–40].

**Fig. 2:**
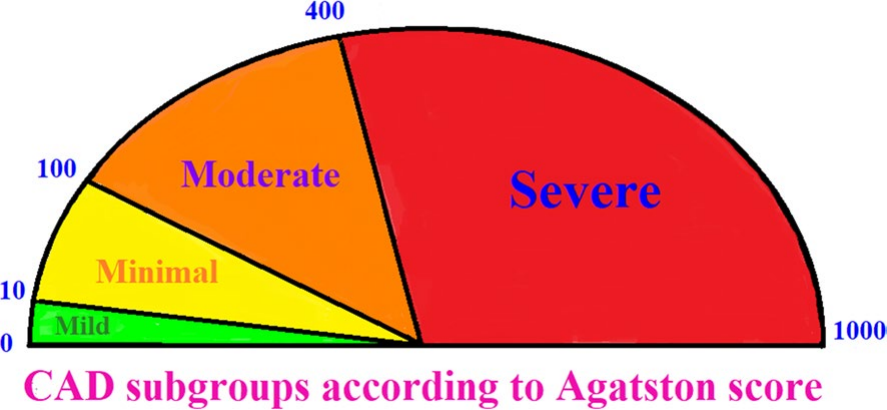
Evaluation of the CAD severity and CAC subgroups according to Agatston score. CAD, coronary artery disease; CAC, calcium score

**Fig. 3:**
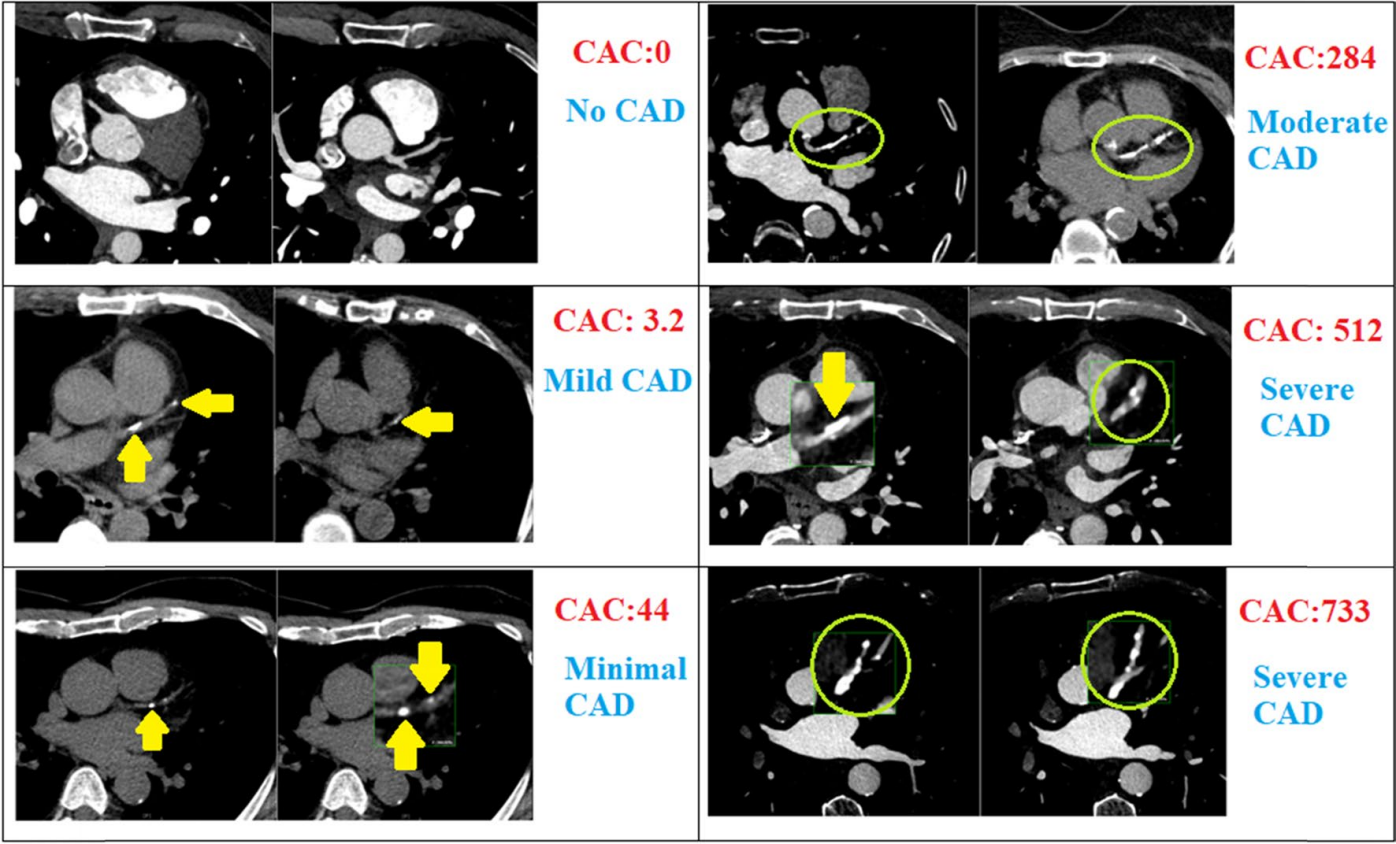
Evaluation of the CAD in patients with assessment of severity in f patients using CAC. CAD, coronary artery disease, CAC, calcium score

Based on their Agatston score, patients are divided into several subgroups: no evidence of CAD (score 0), mild CAD (score 1–10), minimal CAD (score 11–100), moderate CAD (score 101–400), and severe CAD (score > 400) [41]. A growing body of evidence currently considers patients with Agatston scores exceeding 1000 to be a separate subgroup at extreme risk of CVD events, non-CVD outcomes, and mortality [42–45] (Fig. 2,3).

**Fig. 4:**
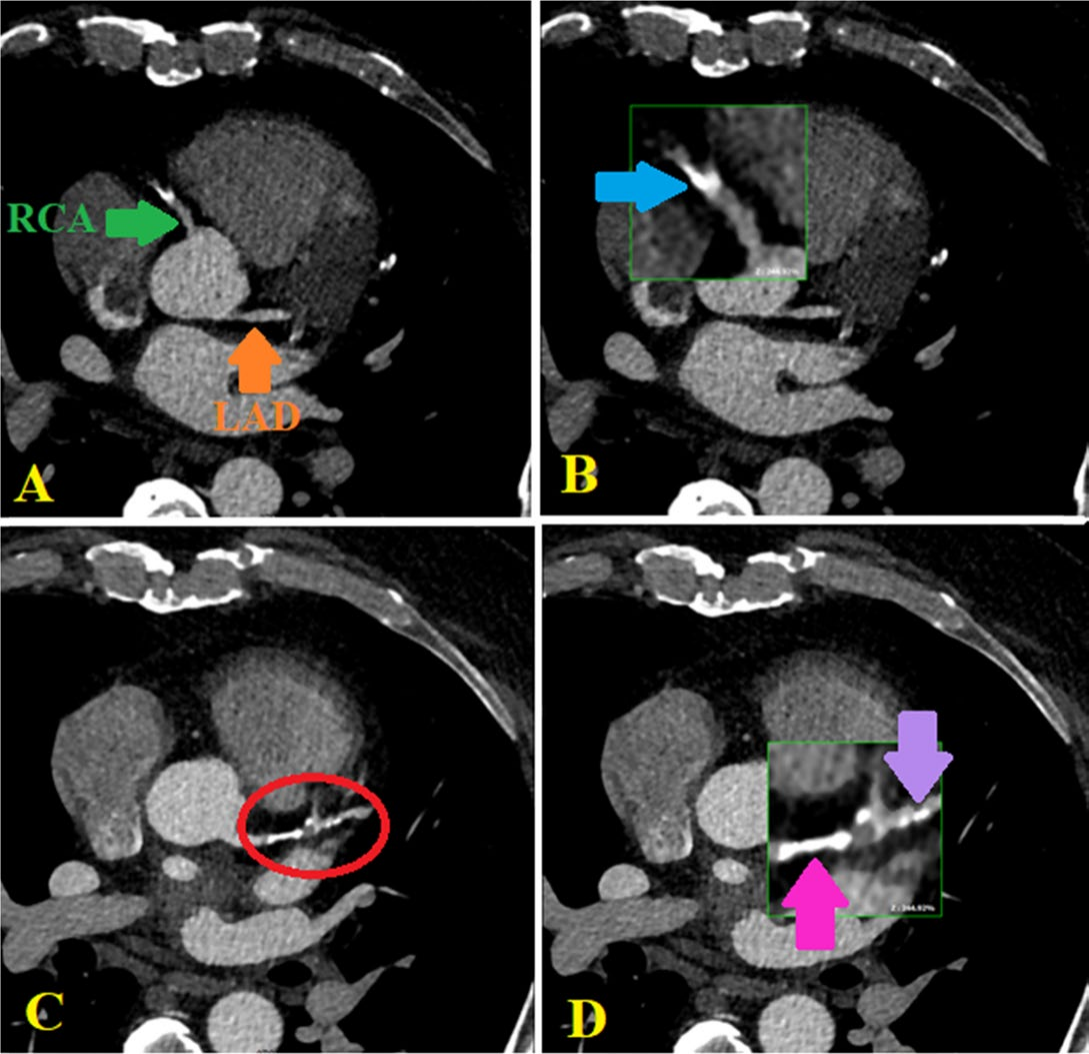
Axial projection of coronary arteries: Diffuse calcification in LAD and RCA in a patient with CAC of 1320. LAD, left anterior descending artery; RCA, right coronary artery; CAD, coronary artery disease; CAC, calcium score

To improve the predictive capabilities of CAC scoring, researchers have introduced various additive factors desirable in the intermediate-risk group, where they can boost decision-making by up-risking or down-risking patients. These factors include the number of vessels with CAC scores above 100, the location of coronary artery calcium, and the number, density, and diffuseness of plaques [46–54] (Fig. 4).

### The ability of the CAC score to predict ASCVD event risks

The net reclassification index (NRI) evaluates risk–prediction instruments [55]. NRI for the CAC score versus FRS is inferred from 3 population-based cohort studies. NRI ranged from 19 to 25% for the whole study population and 52% to 66% for the intermediate-risk group. Additionally, NRI values for other new risk factors, such as the ankle-brachial index, brachial flow-mediated dilation, carotid intima-media thickness, a family history of premature coronary heart disease, and high-sensitivity C-reactive protein, were 3.6%, 2.4%, 10.2%, 165%, and 7.9%, respectively [56–58]. The Multi-Ethnic Study of Atherosclerosis (MESA) reported that the CAC score could predict CVD and coronary heart disease events better than carotid plaque presence or intima-media thickness [59], indicating the substantial superiority of the CAC score in risk assessment over traditional and new risk factor systems [60, 61].

### Clinical guidelines regarding the CT CAC score

The 2019 ACC/AHA Guideline on the Primary Prevention of Cardiovascular Disease recommends CAC measurement as a tool to guide patient-clinician discussion concerning individuals with a 10-year ASCVD risk of 5–7.5% (borderline risk) and those with a 10-year ASCVD risk of 7.5–20% (intermediate risk) [62].

The 2021 Canadian Cardiovascular Society (CCS) Guidelines for the Management of Dyslipidemia for the Prevention of Adult Cardiovascular Disease makes nearly the same recommendations by advising CAC measurement for asymptomatic adults aged 40 or older with a 10–20% ASCVD risk (intermediate risk) based on FRS. The society also recommends CAC scoring for a subset of low-risk adults older than 40 years with a positive family history of premature ASCVD events and genetic propensity for ASCVD [63].

Likewise, the Cardiac Society of Australia and New Zealand (CSANZ) recommends CAC measurement for asymptomatic adults aged between 45 and 75 without a history of CAD and with a 10-year ASCVD risk of 10–20% (intermediate risk), in addition to individuals who have lower 10-year ASCVD risks (6–10%) with a positive family history of premature ASCVD events, and patients aged between 40 and 60 with diabetes [64].

The European Society for Cardiology/European Atherosclerosis Society (ESC/EAS) recommends CAC scoring to decide statin treatment in asymptomatic individuals with low to intermediate risks and advises statin therapy consideration for those with a CAC score above 100 [65].

The National Institute for Health and Care Excellence (NICE) recommends CAC measurement in not only adults at the age of 40 or older, but also for the ones with stable angina after first clinical assessment before CT angiography [66].

To date, the only validated ASCVD risk model to use the CAC score is the MESA risk score, whose predictive value is significantly augmented with the incorporation of CAC scoring [67]. Blaha et al. [68] showed that in the borderline-to-intermediate-risk group, the MESA score with CAC had a substantially better predictive value than that without CAC. The authors also reported that PCE predictions became more robust when the CAC score was added to the equation.

### CAC in specific subgroups

Some studies have shown the ability of the CAC score to predict the outcome of patients with baseline hypertension and underscored its role in this subgroup [69, 70]. On the other hand, a prospective cohort study concluded that individuals with higher CAC scores were more likely to develop hypertension on follow-up [71].

Current smokers are a special subgroup with respect to ASCVD events, and it is essential to consider that a CAC score of zero negative predictive value in this subgroup is not as high as that in other groups. In this regard, Mirbolouk et al. [72] reported double CVD mortality among smokers without CAC compared with their nonsmoking counterparts (sHR, 2.1 and 95% CI, 1.17–3.79). Moreover, McEvoy et al. [73] concluded that smokers with CAC had higher all-cause mortality than smokers without CAC.

Young individuals with underlying diseases, such as systemic lupus erythematosus and chronic kidney disease on hemodialysis, or special psychological traits, such as high hostility levels, loneliness, and social isolation, were prone to accelerated atherosclerotic changes [74–77]. A cohort study on 4182 asymptomatic young adults in the UK noted subclinical atherosclerosis defined as a CAC score > 0 in 20% of the subjects [78]. Kang et al. [79] stated that the presence of CAC in young adults could increase the risk of ASCVD events, with the risk increasing in tandem with a rise in the CAC score.

Chronological dependence of FRS causes false-negative results in younger individuals since they are likely to be labeled low-to-intermediate risk despite the status of other risk factors [80]. Even in young individuals without significant traditional CVD risk factors, a CAC score > 0 is predictive of ASCVD events [81–83], highlighting the need for atherosclerosis screening in the young population. The problem is, however, that screening a large proportion of young people is neither practical nor desirable. In order to address this issue, researchers have demonstrated that initial screening with traditional risk factors can diminish the average number of screened patients to find a CAC-positive individual from 3.5 to 2.2 [83]. In addition, reporting the CAC score on routine chest CT scans can be of value for screening purposes [84].

CVD risk stratification for older individuals via traditional or CAC measurements is challenging because not only does the strong dependency of FRS and PCE on age seemingly increase their risk but also arterial calcification worsens with age advancement [85–87]. Furthermore, the discriminatory value of traditional risk factors in prognosticating ASCVD events is attenuated with advancing age [88]. With the intention of resolving this conundrum, investigators conducted studies and found that the CAC score could identify older individuals with increased risks better than traditional risk factors [89, 90].

Gerke et al. [91–93] developed an age-dependent CAC calculator based on 2 population-based cohort studies (DanRisk and DANKAVAS), which categorized the CAC score according to age and reported that these calculators could make the results of CAC measurement more understandable and applicable for clinical decision-making.

### Two-step measurement approach

Measuring CAC progression as an additional test is a controversial topic. Lehmann et al. [94] compared CAC at baseline and CAC after 5 years to determine whether patients with double-zero results had excellent prognoses concerning 10-year ASCVD events. The authors suggested that a repeated scan within 5 years was beneficial except when the patient already had a double-zero result or, conversely, had a high Agatston score (> 400).

CAC progression is arguably a marker of treatment efficacy, hence its possible beneficial role [41, 95, 96]. Nevertheless, solid evidence is still inadequate to support this claim, and some studies have reported contradictory results showing that after statin initiation, plaque necrotic cores are more likely to undergo calcification and cause CAC score elevations, while plaques will be more stable and less likely to cause ASCVD events [97, 98].

### CAD risk assessment perspective

Non-contrast chest computed tomography scans (NCCTs) are widely employed to detect lung disorders (e.g., infectious, inflammatory, interstitial, and neoplastic). Because lung-cancer screening programs enroll patients with similar cardiovascular risk factors, ASCVD risk assessment is justified. The 2016 guidelines of the Society of Cardiovascular Computed Tomography/Society of Thoracic Radiology (SCCT/STR) recommend qualitative CAC score reporting (none, mild, moderate, and severe) on all NCCTs, regardless of the indication (Class I recommendation)[4].

Artificial intelligence (AI) has emerged as a complementary tool in CAC scoring, and several studies have shown promising results in that regard. Still, some discrepancy exists, warranting further research. Young et al. [99] retrospectively evaluated 452 patient images with both AI-based automated and manual CAC scoring methods and reported that the AI-driven approach enjoyed excellent reliability compared with manual CAC scoring. It should, however, be noted that automated scoring in low-dose CT scans has higher false-positive results than ECG-gated calcium scoring computed tomography. Yu et al. [100] evaluated 405 patients with both non-gated chest CTs and ECG-gated cardiac CTs and concluded that AI algorithms on non-gated chest CT scans per patient could reliably quantify CAC scoring and assign CVD risk categories. Be that as it may, they reported that a slightly significant underestimation of CAC scoring and the regional distribution of CAC without ECG gating were considerable shortcomings.

## Conclusions

Using CAC score measurement is useful and safe for screening of the individuals who are suspicious to coronary artery disease. However, traditional risk assessments tools may prepare valuable data for diagnosis CAD. In conclusion, adding CAC to the traditional risk assessments tools is recommended to evaluate all patient with the risk factors of CAD or the ones who are suspicious to IHD.

## Data Availability

Not applicable.

## Abbreviations

CAD: Coronary artery disease
ASCVD: Atherosclerotic cardiovascular disease
PCE: Pooled cohort equation
BP: Blood pressure
CAC: Coronary artery calcium
FRS: Framingham risk score
ACC/AHA: American College of Cardiology/American Heart Association
CT: Computed tomography
ECG: Electrocardiogram
NRI: Net reclassification index
MESA: Multi-Ethnic Study of Atherosclerosis
CCS: Canadian Cardiovascular Society
CSANZ: Cardiac Society of Australia and New Zealand
ESC/EAS: European Society for Cardiology/European Atherosclerosis Society
NICE: National Institute for Health and Care Excellence
NCCTs: Non-contrast chest computed tomography scans
SCCT/STR: Society of Cardiovascular Computed Tomography/Society of Thoracic Radiology
AI: Artificial intelligence
